# Psychological pain treatment in fibromyalgia syndrome: efficacy of operant behavioural and cognitive behavioural treatments

**DOI:** 10.1186/ar2010

**Published:** 2006-07-19

**Authors:** Kati Thieme, Herta Flor, Dennis C Turk

**Affiliations:** 1 Department of Clinical and Cognitive Neuroscience, University of Heidelberg, Central Institute of Mental Health, J5, 68169 Mannheim, Germany; 2 Department of Anesthesiology, University of Washington, 1959 NE Pacific Street, Box 356540, Seattle, Washington 98195-6540, USA

## Abstract

The present study focused on the evaluation of the effects of operant behavioural (OBT) and cognitive behavioural (CBT) treatments for fibromyalgia syndrome (FMS). One hundred and twenty-five patients who fulfilled the American College of Rheumatology criteria for FMS were randomly assigned to OBT (*n *= 43), CBT (*n *= 42), or an attention-placebo (AP) treatment (*n *= 40) that consisted of discussions of FMS-related problems. Assessments of physical functioning, pain, affective distress, and cognitive and behavioural variables were performed pre-treatment and post-treatment as well as 6 and 12 months post-treatment. Patients receiving the OBT or CBT reported a significant reduction in pain intensity post-treatment (all Fs > 3.89, all *P*s < 0.01). In addition, the CBT group reported statistically significant improvements in cognitive (all Fs > 7.95, all *P *< 0.01) and affective variables (all Fs > 2.99, all *P*s < 0.02), and the OBT group demonstrated statistically significant improvements in physical functioning and behavioural variables (all Fs > 5.99, all *P*s < 0.001) compared with AP. The AP group reported no significant improvement but actually deterioration in the outcome variables. The post-treatment effects for the OBT and CBT groups were maintained at both the 6- and 12-month follow-ups. These results suggest that both OBT and CBT are effective in treating patients with FMS with some differences in the outcome measures specifically targeted by the individual treatments compared with an unstructured discussion group. The AP group showed that unstructured discussion of FMS-related problems may be detrimental.

## Introduction

Fibromyalgia syndrome (FMS) is defined by the presence of widespread pain of at least 3 months' duration and pain upon palpation of at least 11 out of 18 specific tender points (TPs). Patients diagnosed with FMS also report disordered sleep, excessive fatigue, and a range of physical [1,2], cognitive [3,4], affective [5,6], stress-related [7,8], and behavioural symptoms [9,10]. The cause of FMS is not known; however, several mechanisms may be involved [11-13].

Two psychologically based treatment approaches, cognitive behaviour therapy (CBT) and operant behaviour therapy (OBT), have been reported to provide benefits for a significant proportion of patients with FMS [14-16]. A meta-analysis [17] of 49 treatment outcome studies compared the efficacy of pharmacological and non-pharmacological treatments. CBT yielded significantly greater improvements in physical status, symptoms, psychological functioning, and functional ability compared with physical therapy and was more effective for FMS symptoms and daily functioning than was pharmacological treatment with antidepressants [17]. These results and a recent evidence-based clinical practice guideline [18] suggest that optimal treatment of FMS should include physical exercise, antidepressant medication, and cognitive behavioural methods.

Treatment based on operant conditioning [19] has been applied to a variety of chronic pain syndromes. OBT emphasises increased activity, inclusion of significant others to reduce reinforcement of pain behaviours, and the reduction of pain-contingent medication [19,20]. Only a few studies have reported on the effectiveness of OBT with patients with FMS [14,21]. For example, OBT was shown to produce a significant and stable reduction in pain intensity, interference, solicitous behaviour of the spouse, medication, pain behaviours, number of physician visits and days at a hospital, and improvement in sleeping. Sixty-five percent of the OBT-treated patients showed clinically significant improvement when compared with patients who received physical therapy alone [21].

Although OBT and CBT share some common elements, they make different assumptions and have different emphases. OBT focuses on the modification of reinforcement contingencies that maintain pain behaviours and on changing pain-related behaviours, whereas CBT emphasises the role of maladaptive beliefs and expectations of patients (that is, cognitive variables in the maintenance and exacerbation of symptoms and disability) and thus aims primarily to alter the attitude of the patients toward the pain and self-management.

Turk and colleagues have demonstrated that patients diagnosed with FMS are heterogeneous [13], characterised by several patterns based on how they respond to their symptoms. They suggested that different treatments that are matched to specific psychosocial and behavioural features may be required. The aims of the present study were (a) to examine the effectiveness of CBT and OBT in comparison with an attention-placebo (AP) group and (b) to compare the relative effectiveness of OBT and CBT with each other.

### Specific hypotheses

1. CBT and OBT will produce significant improvements in pain, physical functioning, and emotional distress in patients with FMS.

2. CBT and OBT will produce significantly greater improvements in pain, physical functioning, and emotional distress than the AP treatment.

3. CBT will produce significantly greater effects than the OBT and AP groups on coping and catastrophising responses.

4. OBT will produce significantly greater reductions in pain behaviours, physical disability, and physician visits than the CBT or AP treatments.

## Materials and methods

### Participants

A sample of 125 consecutive married female patients with FMS was recruited from 10 outpatient rheumatological clinics. The groups were comparable with respect to demographic and FMS-specific variables (for example, number of TPs and severity of TP pain [22]; Table 1).

### Study protocol

**Figure 1:**
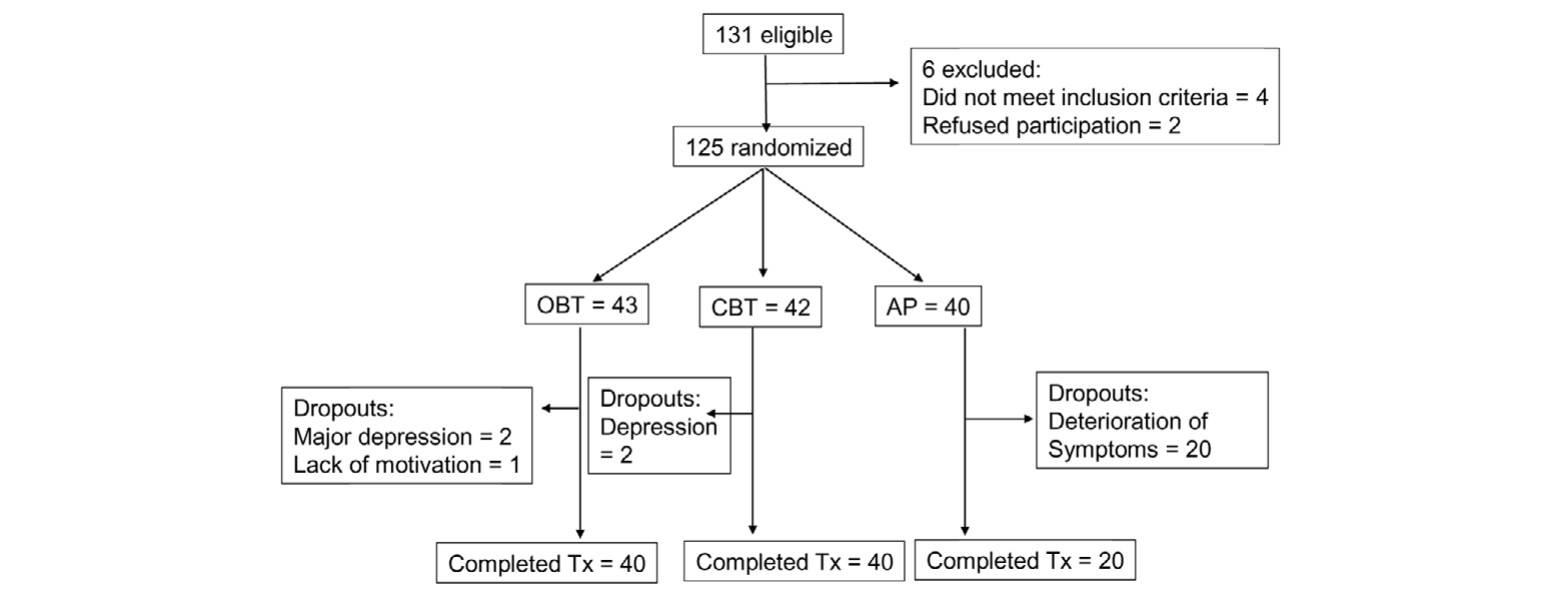
CONSORT (Consolidated Standards for Reporting of Trials) diagram. AP, attention-placebo; CBT, cognitive behavioural therapy; OBT, operant behavioral therapy; Tx, treatment.

All patients signed informed consent and were randomly assigned to OBT, CBT, or AP treatment. The study was approved by the local ethics committee. The administering of the three types of treatment was counterbalanced in order to control for time of year and time of entry into the clinical trial. Figure 1 provides an overview of the patient flow in the study based on the CONSORT guidelines [23].

All patients received a general medical and a rheumatological assessment (see below). The inclusion criteria consisted of (a) meeting ACR (American College of Rheumatology) criteria of FMS [1], (b) pain for a period of at least 6 months, (c) married, (d) willingness of the spouse to participate, and (e) ability to complete the questionnaires and understand the treatment components. The exclusion criteria consisted of inflammatory rheumatologic diseases and any concurrent major disease such as cancer, diabetes, or kidney failure.

### Assessments

#### Physical assessment

The physical assessment included blood chemistry analysis, neurological examination, and evaluation of TPs. The number of positive TPs and pain intensity of TPs, rated on a numeric scale from 0 (no pain) to 10 (worst pain possible), were assessed using the Manual Tender Point Survey [23], and responses were calculated by summing the patients' responses to palpation of the 18 TPs.

#### Psychometric assessment

Three self-report measures that had been used in previous studies of FMS (for example, [6,8,21]) were included. These consisted of the following:

The Fibromyalgia Impact Questionnaire (FIQ) [24,25] is a 19-item self-report questionnaire measuring physical impairment, fatigue, stiffness, and functional activities, including sleep. The FIQ has good psychometric properties (for example, [26]).

The West Haven-Yale Multidimensional Pain Inventory (MPI) [27,28] is a 60-item questionnaire assessing pain intensity, interference of pain, life control, affective distress, social support, significant-other responses, and general activity levels. The MPI has been widely used with diverse chronic pain samples, including FMS [28-30], and has been demonstrated to have good psychometric properties [28-30].

Cognitive variables were assessed using the 32-item Pain-Related Self-Statements Scale (PRSS) [31] with the subscales 'active coping' (for example, 'I can handle my pain') and 'catastrophising' (for example, 'I am a hopeless case') shown to have excellent psychometric properties [31].

In addition to performing the measures enumerated above, all patients completed treatment expectation ratings before the first sessions and satisfaction ratings at the end of the first and last sessions based on Borcovec and Nau [32]. Satisfaction was rated on a 6-point scale ranging from 0 ('completely unsatisfied') to 6 ('completely satisfied'). This measure was included as a means of determining whether the groups differed in their beliefs about the quality of the treatment received.

#### Assessment of pain behaviors

Pain behaviours were elicited by the standardised performance of a window washing task. Patients were videotaped performing this task. Pain behaviours were assessed using the Tübingen Pain Behaviour Scale (TBS) [33]. The frequency of occurrence of each pain behaviour was coded by two independent raters in 10-second epochs for a period of 8 minutes [10]. The TBS rates the presence of behaviours on a 0–2 scale (0 = none, 1 = sometimes, 2 = always). The total value of pain behaviours was calculated by summing the absolute frequencies of the individual pain behaviours observed during the task. The inter-rater reliability was good (kappa = 0.82; *P *< 0.001). Scores for pain behaviours in the absence and in the presence of the spouse were computed.

#### Health care utilisation

Medication consumption and number of physician visits 12 months prior to and 12 months after treatment were obtained from the medical records maintained at the various Rheumatology Outpatient Clinics. Patients were routinely observed at the clinic at 6-week scheduled intervals.

### Treatments

Each treatment consisted of 15 weekly 2-hour sessions co-led by a psychologist and rheumatologist and conducted in groups of five patients. Spouses attended the first, fifth, ninth, and 13th sessions. Both CBT and OBT were based on structured manuals [34], whereas the AP treatment consisted of unstructured discussions of problems associated with having FMS and support provided by the therapists and group members.

#### Cognitive behavior therapy

The CBT focused on the patients' thinking and involved problem-solving, stress and pain coping strategies, and relaxation [34,35]. Patients were taught the meaning of the stress-tension-pain circle as a cognitive pain model and learned coping strategies and the reduction of catastrophising thoughts. Patients and spouses received weekly homework tasks, were encouraged to engage in physical activities, and were asked to reduce analgesic medication use at a gradual rate over the course of the treatment. The patients participated in relaxation exercises during and between the sessions. The therapists identified instances of maladaptive thinking and encouraged the group to challenge these instances and to provide more appropriate interpretations and alternatives. Although the importance of behaviour change was noted, the focus of this treatment was on the change of maladaptive thoughts and attitudes. The treatment was administered in the fashion of a Socratic dialogue.

#### Operant behavior therapy

The OBT was primarily based on changing observable pain behaviours and included video feedback of expressions of pain as well as contingent positive reinforcement of pain-incompatible behaviours and punishment of pain behaviours in a group setting. Structured time-contingent exercises were provided according to operant principles [20] in the sessions and as homework exercises. The treatment also included time-contingent intake and reduction of medication, increase of bodily activity, reduction of interference of pain with activities, reduction of pain behaviours, and training in assertive pain-incompatible behaviours [34]. Patients engaged in role-playing to reduce pain behaviours and increase healthy behaviours. Patients, their spouses, as well as group members used a reinforcer plan that consisted of the presentation of a 'red card' when pain behaviours were displayed and a 'green card' when healthy behaviours were displayed. Patients were encouraged to increase their activity levels and were assigned homework that included specific instructions to increase activities and reduce pain behaviours. A reduction of medication was instituted immediately after the assessment phase, based on a physician-coordinated individual time-contingent interval plan. In contrast to CBT, this treatment focused primarily on behavioural expressions of pain and emphasised changing inappropriate pain behaviours without directly targeting maladaptive thoughts or cognitive aspects of coping.

#### Attention placebo

The AP treatment focused on general discussions among patients in groups guided by therapists. The discussions were centered around medical and psychosocial problems of FMS (that is, stress in different areas of the patients' lives, physician-patient interaction, and use of medication). Within the groups, patients were provided with opportunities to speak about problems with coping, fatigue, pain, stress, and medication. The therapists did not initiate these topics and made no specific recommendations. The patients did not receive any specific homework.

#### Treatment adherence

Adherence to the treatment was assessed by the number of sessions attended and the completion of homework assignments in CBT and OBT (Table 1).

#### Therapists

Three psychologists, each with more than 15 years of experience of treatment, conducted the groups. They completed a 2-day training program together with 10 rheumatologists who served as co-therapists. Additionally, psychologists and rheumatologists met to decide which study information the patients should receive from the physician and to outline strategies for difficult situations, including problems with motivation and non-adherence.

### Statistical analyses

The intent-to-treat principle guided the analyses such that the baseline scores (that is, 'last' observation) for those who terminated treatment prematurely were carried forward. The primary outcome measures were changes in pain intensity, physical functioning, affective distress, and health care utilisation [23,36] at post-treatment and the 6- and 12-month follow-ups. The initial analyses of treatment effectiveness were assessed using a multivariate analysis of variance (MANOVA) for pain, function, and mood. Significant main effects and interactions were followed by post hoc analysis of variance (ANOVA) and *t *tests.

The emphasis of CBT was on changing beliefs and expectancies, whereas the OBT was designed to change pain behaviours. To confirm the validity of the treatments, a series of 2 × 2 MANOVAs was performed. Significant effects were followed up with univariate ANOVAs and *t *tests. The outcome MANOVA included three variables and used a *P *value Bonferroni-adjusted to *P *< 0.02. The MANOVA on cognitive and behavioural effects included two variables and used a Bonferroni-adjusted *P *< 0.03.

To determine whether the treatment effects were clinically significant, the effect sizes (ESs) for the combined OBT and CBT groups and the individual responses for the OBT and CBT groups were compared with the AP group and computed based on the formula: AP (mean_T2–4_) – CBT [or OBT] (mean_T2–4_)/CBT/[or OBT] (standard deviation_T1_) [37]. To avoid overestimation of the CBT and OBT related to the deterioration of the AP group which became apparent during data analysis, ESs were compared for the entire AP group and the subgroup of AP patients who dropped out and whose values were carried forward. This procedure compared the ESs of the OBT and CBT groups with a no-change subgroup of the AP group. The comparison of CBT and OBT with the AP group may have been distorted by the large number of dropouts in the AP group and the deterioration in many variables in the patients who remained in the group. Therefore, we computed an adjusted ES that included the baseline values of the dropouts of the AP group which were carried forward for the post-treatment analyses at the 12-month follow-up.

## Results

### Attrition

Three patients in the OBT (6.9%), two in the CBT (4.8%), and 20 in the AP (50%) groups terminated the treatment prematurely (Figure 1). All dropouts occurred between sessions 1 and 4. The primary reason that patients gave for dropping out of the AP group was deterioration of symptoms. Patients who terminated prematurely were not significantly different from those who completed treatment in duration of symptoms, initial pain severity, or number or severity of TPs. Overall, 100 patients completed the treatments, 40 in the OBT group, 40 in the CBT group, and 20 in the AP group.

### Treatment expectation and satisfaction

There were no statistically significant differences between the groups in treatment expectations (F(2, 122) = 1.47, *P *= 0.24) in the first treatment session. For treatment satisfaction, calculated as a combination of first and last session, an ANOVA revealed neither a significant group (F(2, 122) = 1.42, *P *= 0.25) nor a significant group × phase (first versus last session) (F(2, 122) = 0.53, *P *= 0.59) effect.

The adherence of patients in the CBT and OBT groups was excellent. In the OBT group, only 4.3% sessions were missed and 5.5% of homework was not completed. In the CBT group, 3.2% sessions were missed and 4.7% of homework was not completed. The subsample of the AP group who were retained in the treatment missed 7.6% sessions.

### Primary outcomes

Physical impairment was assessed by the FIQ, and pain intensity and affective distress were assessed by the MPI. Number of physician visits was used as behavioural variable [35,36].

The MANOVA revealed a significant effect of both group (F(2, 122) = 15.63, *P *< 0.001) and outcome (F(3, 120) = 82.53, *P *< 0.001) variables. There was no significant effect of time but significant time × group (F(2, 122) = 15.92, *P *< 0.001), outcome variables × time (F(3, 120) = 4.79, *P *< 0.005), and group × time × outcome variables (F(3, 121) = 12.53, *P *< 0.001) interactions. The post hoc ANOVA revealed a significant difference between CBT and AP (*P *< 0.001) and between OBT and AP (*P *< 0.001) but not between CBT and OBT (*P *= 1.00).

### Physical impairment

**Figure 2:**
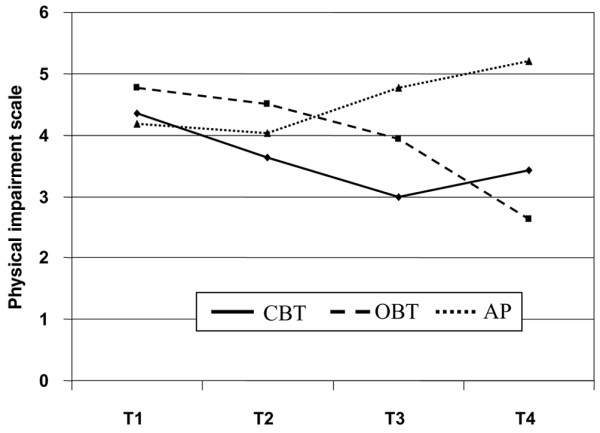
Differences in physical impairment among cognitive behavioural therapy (CBT) (solid line), operant behavioral therapy (OBT) (dashed line), and attention-placebo (AP) (dotted line) groups prior to treatment (T1), immediately after treatment (T2), and at 6- (T3) and 12-month (T4) follow-ups.

The post hoc ANOVA revealed a statistically significant group × time interaction (Table 2) with OPT and CBT significantly different from AP but not from one another. Interestingly, OBT and CBT showed a statistically significant decrease of functional limitations at the 12-month follow-up, whereas the AP group displayed a statistically significant increase at the 12-month follow-up (Figure 2). However, only the OBT showed significantly reduced functional limitations 12 months after the treatment compared with pre-treatment (Table 2) whereas their functional limitations did not change immediately after or 6 months after treatment. Functional limitations showed a large ES (Table 3) for the OBT (1.15) which increased from pre-treatment to the two follow-up periods.

### Pain intensity

**Figure 3:**
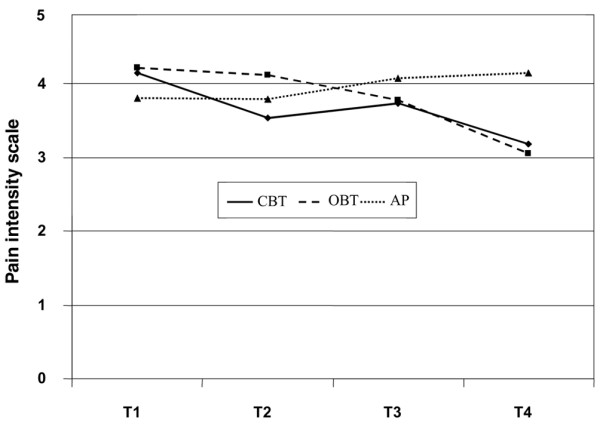
Differences in pain intensity among cognitive behavioural therapy (CBT) (solid line), operant behavioral therapy (OBT) (dashed line), and attention-placebo (AP) (dotted line) groups prior to treatment (T1), immediately after treatment (T2), and at 6- (T3) and 12-month (T4) follow-ups.

The ANOVA on pain intensity revealed a significant group × time interaction (F(3, 121) = 11.95, *P *< 0.001) with significant differences between AP and both CBT and OBT. Both CBT and OBT showed significant pain reduction (Table 2) at the 6-month and 12-month follow-ups. Unexpectedly, the AP group showed a statistically significant increase of pain intensity 6 months after the treatment in comparison with the CBT and OBT. There were no significant differences between CBT and OBT at the 6-month and 12-month follow-ups (Figure 3). Comparable with physical impairment, pain intensity did not change immediately after or 6 months after treatment (Table 2). There were large ESs for pain intensity (Table 3) in both the CBT (1.14) and the OBT treatments (1.10). In general, the ESs of the CBT and OBT increased over time, supporting the maintenance of the improvements.

### Affective distress

Consistent with the results for pain and functional impact, the ANOVA yielded a significant group × time interaction (F(3, 121) = 5.89, *P *< 0.005) with significant group differences between CBT and AP and between OBT and AP but not between CBT and OBT (Table 2). The CBT showed a significant decrease of affective distress (Table 2) immediately after, 6 months after, and 12 months after treatment. The OBT did not display any significant changes over time (Table 2). In contrast to CBT and OBT (Table 2), the AP group showed an increase of affective distress at 6 months after treatment and a further increase 12 months after treatment.

**Figure 4:**
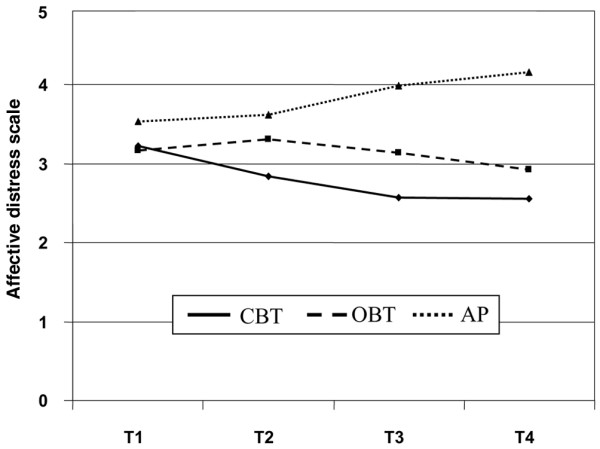
Differences in affective distress among cognitive behavioural therapy (solid line), operant behavioral therapy (dashed line), and attention-placebo (dotted line) groups prior to treatment (T1), immediately after treatment (T2), and at 6- (T3) and 12-month (T4) follow-ups.

In comparison with AP, the CBT patients achieved significantly lower levels of affective distress immediately after, 6 months after, and 12 months after treatment, the OBT patients only after 12 months. There were no significant differences between CBT and OBT (Figure 4). The CBT demonstrated large effects in reducing affective distress (0.76–1.57) with increasing ESs over time (Table 3).

### Physician visits

An ANOVA demonstrated a significant group × time interaction (F(2, 122) = 33.52, *P *< 0.001) with significant group differences between OBT and AP (*P *< 0.001) and between CBT and AP (*P *= 0.001) but not between CBT and OBT (*P *= 0.48). The CBT did not show any significant changes over time (Table 2). The OBT displayed a significant decrease of physician visits (Table 2) at the 12-month follow-up, whereas the AP group showed a significant increase of the numbers of physician visits at the 12-month follow-up.

In comparison with AP patients, CBT- and OBT-treated patients had made significantly fewer physician visits at the 12-month follow-up. There were, however, no significant differences between CBT and OBT. The number of physician visits showed a very large ES for the OBT with 2.13 (1.45 adjusted).

### Targeted treatment effects

#### Cognitive variables

The CBT treatment was specifically designed to target maladaptive beliefs. To confirm the efficacy of the treatment on cognitive variables, a MANOVA on the two scales of the PRSS, active coping and catastrophising, was calculated. It revealed a significant effect of time (F(1, 122) = 4.52, *P *< 0.03), cognitive variables (F(1, 122) = 5352, *P *< 0.001), cognitive variables × time (F(1, 122) = 8.32, *P *< 0.01), cognitive variables × group (F(2, 122) = 14.92, *P *< 0.001), and cognitive variables × time × group (F(2, 122) = 27.41, *P *< 0.001). There was no significant effect of treatment (F(1, 122) = 0.77, *P *= 0.47).

##### Coping

An ANOVA demonstrated a significant interaction for group × time (F(3, 121) = 16.18, *P *< 0.001) with significant group differences between CBT and AP and between OBT and AP but not between CBT and OBT. Both CBT and OBT showed a significant increase of adaptive coping (Table 4) immediately after the treatment as well as at the 6-month and 12-month follow-ups. In contrast to CBT and OBT (Table 4), the AP group showed a decline in the use of positive coping at both follow-ups.

In comparison with the AP, the CBT-treated patients responded with improved coping immediately after, 6 months after, and 12 months after the treatment, OBT-treated patients only after 6 and 12 months. There were no significant differences between CBT and OBT. CBT produced a larger ES (2.66) on active coping than did the OBT treatment (1.23), although both were large. In general, the ESs of the CBT and OBT increased over time (Table 3).

##### Catastrophising

Post hoc ANOVAs demonstrated a significant treatment × time interaction (F(3, 121) = 12.99, *P *< 0.001) with significant group differences between CBT and AP (*P *< 0.03) and between OBT and AP (*P *< 0.005) but not between CBT and OBT. The CBT and OBT showed a significant decrease of catastrophising (Table 4) immediately after treatment as well as at the 6- and 12-month follow-ups. In contrast to the CBT and OBT (Table 4), the AP group showed an increase of catastrophising at the follow-ups.

The CBT and OBT groups showed significantly less catastrophising immediately after treatment, and this was maintained over both follow-ups in comparison with the AP group. Larger ESs for catastrophising were obtained for CBT (1.44) than for OBT (0.97), although both ESs were large (Table 3).

#### Behavioural variables

These included pain behaviours and pain-related solicitous spouse behaviours. A MANOVA revealed a significant effect for group (F(2, 122) = 3.36, *P *< 0.03), variables (F(2, 121) = 126.35, *P *< 0.001), variables × group (F(2, 121) = 9.12, *P *< 0.001), time × group (F(2, 122) = 16.39, *P *< 0.001), and group × time × variables (F(2, 122) = 30.09, *P *< 0.001). Post hoc analyses showed a significant difference between OBT and AP (*P *< 0.02) but not between CBT and AP or between CBT and OBT.

##### Pain behaviors

Post hoc ANOVAs demonstrated a significant treatment × time (F(3, 121) = 11.39, *P *< 0.001) effect with significant group differences only between OBT and AP (*P *< 0.02) but not between CBT and AP or between CBT and OBT (Table 4). Whereas the CBT did not show any significant decrease of pain behaviour (Table 4), the OBT displayed a significant decrease of pain behaviour (Table 4) immediately after the treatment and at the 6-month and 12-month follow-ups. In contrast to CBT and OBT (Table 4), the AP group showed a significant increase in pain behaviours immediately after and at the 6- and 12-month follow-ups.

OBT patients reduced their pain behaviours immediately after the treatment, an effect that was maintained for a period of at least 12 months. CBT patients showed a significant reduction in pain behaviours only 12 months after treatment. Overall, observed pain behaviours showed a very large ES (Table 3) for OBT (1.59) in contrast to a moderate ES for CBT (0.57).

##### Significant-other behaviours

The ANOVA revealed a significant group × time interaction (F(3, 121) = 7.65, *P *< 0.001) with significant group differences between OBT and AP (*P *< 0.02) but not between CBT and AP or between CBT and OBT. The OBT displayed a statistically significant decrease of solicitous spouse behaviour immediately after the treatment as well as at the two follow-ups. The AP group showed a statistically significant increase of solicitous behaviour at the follow-ups. The CBT did not show any significant changes over time (Table 4). The CBT patients showed lower solicitous spouse behaviour immediately after the treatment in comparison with the AP patients. However, this effect was not maintained. The OBT group showed fewer solicitous spouse behaviours than the AP patients at the 12-month follow-up. There were no significant differences between CBT and OBT. Only the OBT experienced a significant reduction of solicitous significant-other behaviours over time. The ES for reduced solicitous spouse behaviour (Table 3) after OBT increased over the time.

##### Adjusted ESs

The adjusted ESs were still moderate to high but substantially lower than the uncorrected ESs (Table 3). The OBT showed greater effects in the reduction of physical impairment (ES = 1.07) and pain behaviour (ES = 1.48), whereas the CBT showed the greatest increase in coping (ES = 1.70) and reduction in affective distress (ES = 0.61).

## Discussion

Both the CBT and OBT groups reported significant improvements in physical functioning, pain, and emotional distress 1 year after treatment, in comparison with the AP group. The latter actually demonstrated significant deterioration after treatment. Even though no statistically significant differences were identified favoring CBT or OBT overall, the within-calculations for each group over time revealed that the CBT did not demonstrate as pronounced a set of treatment effects in functional limitations, whereas the OBT revealed somewhat less of a treatment effect in affective distress.

A clear superiority was found for the active psychological interventions in comparison with the AP group. In fact, the results were increased at the 6-month and 12-month follow-ups, and notably, the demonstrated beneficial effects were achieved without the inclusion of an additional structured physical therapy program or additional antidepressant medication. These results have important implications because physical therapy and antidepressant medication are often recommended as important components of treatment for FMS [18]. Future studies should directly compare these very different treatment approaches and also perform responder analyses to clarify the characteristics of patients who require different treatments to achieve beneficial effects.

Interestingly, no significant differences on the cognitive variables were observed between the CBT and OBT groups. One explanation that seems plausible is that, although the CBT treatment directly focused on cognitive variables, it is possible that the behavioural changes and symptom improvements achieved by the patients treated by OBT produced changes in active coping and catastrophising without targeting those directly. Thus, observation of one's behaviour and experiences of increased activities may produce changes in a patient's beliefs about their plight.

The most significant changes for CBT were found with respect to pain and cognitive and affective variables. Positive cognitions were successively increased, and patients learned to improve their use of coping strategies to decrease catastrophic thinking with the consequence of reduced affective distress. These results are consistent with previous research [15,16] and indicate that the treatment successfully targeted improvements in cognitive coping. These results were stable over 12 months and clinically significant. Despite the fact that the CBT treatment did not directly focus on pain behaviours, the present results support a moderate benefit of the treatment on behaviours (ES = 0.57, 0.49 adjusted). Apparently, changing patients' beliefs is a critical aspect of treatment, regardless of whether they are directly targeted or derived from the observation of the patients' own behaviour [38].

Significant changes for OBT were found with respect to pain and physical and behavioural variables. In accordance with previous reports [14,21], healthy behaviours were successively increased and pain behaviours were decreased. The OBT, notably, achieved statistically significant reductions in physician visits (50%) in direct contrast to the AP group, which almost doubled the number of visits. CBT, however, produced only a modest and not statistically significant reduction of physician consultations. These results suggest that the OBT treatment may not only provide clinical benefits but also produce significant reductions in health care utilisation.

As hypothesised, the analysis of clinical significance demonstrated that CBT had a relatively greater effect in the reduction of affective distress and catastrophising, whereas OBT had a relatively greater effect in the reduction in functional limitations, pain behaviours, and solicitous spouse behaviour. These data support the validity of the treatments. Regarding pain intensity and coping, CBT and OBT showed similar effects. CBT focused on changes of cognitions with the effect of reduced cognitive factors of pain, whereas OBT focused on behavioural changes and reached reductions of physical and operant components of pain. Although it is not surprising that CBT and OBT reached comparable effects in pain reduction, the focus of treatment used to accomplish these outcomes varied substantially. Taken together, these data suggest that CBT might be especially beneficial in patients with high levels of cognitive maladaptation to the pain and high affective distress, whereas OBT might be especially efficacious in patients with high levels of pain behaviours and low physical functioning. Future research will have to determine to what extent this is true or whether it might be beneficial to combine elements of both treatments.

Unexpectedly, the AP group significantly deteriorated during the study, with a worsening of symptoms on all outcome measures, which may explain the large proportion (50%) of AP patients' terminating treatment prematurely. Unstructured discussion about problems aligned with coping with chronic pain appeared to lead to increased pain, functional limitations, emotional distress, and pain behaviours. This might be due to the disease-oriented, solicitous behaviour of group members which may have reinforced pain and pain behaviour. The deterioration of the AP was not limited to the pre-post comparison but persisted up to 12 months after treatment termination. One possible explanation for the large dropout in the AP group might be that the treatment was simply not credible or was viewed as unsatisfactory. This explanation was not supported by the results on patient satisfaction. There were no significant differences in patient satisfaction among the three groups. The long-lasting deterioration of the AP group was unexpected and needs to be explored. If confirmed, these results suggest that, at least for a subset of patients, discussions about pain and possibly informal support groups may be detrimental.

There are several limitations in this study, and the large number of dropouts in the AP group is disconcerting indeed. The unexpected detrimental effects of the AP group are a significant concern and may have contributed to the large ESs reported. Because this result may have overestimated the true effects of the behavioural treatments, we also computed ESs for the carried-forward baseline data of the dropouts. The ESs that were obtained are still moderate to large, suggesting a substantial effect of the behavioural treatments.

The interpretation of the results of the ANOVAs on the subgroups must be considered with caution due to the small sample sizes. Larger studies are needed to replicate the results. All participants in this study had to have a spouse who was willing to participate. Turk *et al*. [13] reported that almost 40% of patients with FMS indicated high levels of interpersonal distress. The generalisability of the results of this study to unmarried FMS patients and patients with poor interpersonal relations also needs to be confirmed.

## Conclusion

Despite the limitations noted, it appears that psychologically based treatments are capable of producing both statistically significant and clinically meaningful benefits for patients with long-standing FMS, without the specific inclusion of a structured physical therapy plan or antidepressant medications. Importantly, the results demonstrated that the changes were maintained up to 12 months after treatment. Moreover, there were significant reductions in physician visits, primarily for the OBT treatment, that would result in substantial cost savings in response to FMS therapy. Although promising, these treatments are geared toward FMS symptoms such as pain, physical impairment, and affective distress but probably do not address causal factors of the disorder, which are still unknown.

## Abbreviations

ANOVA = analysis of variance; AP = attention-placebo; CBT = cognitive behavioural therapy; ES = effect size; FIQ = Fibromyalgia Impact Questionnaire; FMS = fibromyalgia syndrome; MANOVA = multivariate analysis of variance; MPI = West Haven-Yale Multidimensional Pain Inventory; OBT = operant behavioral therapy; PRSS = Pain-Related Self-Statements Scale; TBS = Tübingen Pain Behaviour Scale; TP = tender point.

## Competing interests

The authors declare that they have no competing interests.

## Authors' contributions

KT provided the design of the study, recruitment of the patients, organisation and realisation of the experimental design, statistical analyses, interpretation of the results, and preparation of the manuscript. HF provided the design of the treatments, supervision of data analyses, interpretation of the results, and preparation of the manuscript. DCT provided advice on the statistical analysis, interpretation of the results, and preparation of the manuscript. All authors read and approved the final manuscript.

## Figures and Tables

**Table 1:** Demographic and clinical data of the patients (*n *= 125)

	OBT (*n *= 43)	CBT (*n *= 42)	AP (*n *= 40)
	Mean SD (Range)	Mean SD (Range)	Mean SD (Range)
Age (in years)	43.23	49.13	47.46
	9.03	10.03	9.75
	(21–59)	(22–66)	(21–67)
Duration of pain (in years)	8.98	9.08	8.73
	10.11	8.50	8.77
	(1.5–43)	(0.5–36)	(0.5–43)
Number of painful regions	7.41	6.74	7.06
	1.81	2.18	2.07
	(4–10)	(3 – 10)	(3–10)
Number of tender points	16.66	17.25	16.88
	3.93	4.47	4.33
	(11–18)	(11–18)	(11–18)
Mean tender point pain intensity	5.73	4.59	4.21
	2.14	1.49	1.73
	(1.7–8.3)	(1.9–7.3)	(0–10)
Number of physician visits	36.87	30.55	34.25
	15.15	16.20	16.33
	(18–69)	(4–86)	(14–84)
Drug (*n*/day of amytriptiline 25 mg)	3.62	3.16	3.36
	2.16	3.62	2.78
	(0–9)	(0–16)	(0–12)
Treatment expectation	4.33	4.30	4.06
	1.17	1.07	0.99
	(2–6)	(2–6)	(2–6)
Treatment satisfaction (first session)	4.39	4.30	3.94
	0.99	0.81	0.87
	(3–6)	(3–5)	(3–5)
	*n *(%)	*n *(%)	*n *(%)

Occupational status			
Working	17 (39.5)	19 (45.2)	20 (50.0)
Unemployed	16 (37.2)	16 (38.1)	12 (30.0)
Workers' compensation	3 (7.0)	2 (4.8)	3 (7.5)
Retired	7 (16.3)	4 (9.5)	3 (7.5)
Student	0 (0.0)	1 (2.4)	2 (5.0)

AP, attention-placebo; CBT, cognitive behavior therapy; OBT, operant behaviour therapy; SD, standard deviation.

**Table 2:** Means, SDs, and F and *P *values for ANOVA effects for group, time, and group × time (G × T) and T and *P *values for the main outcome variables

Outcome variables											
						Main effects					
Outcome variables	Group	Pre-treatment Mean (SD)	Post-treatment Mean (SD)	6-month f/u Mean (SD)	12-month f/u Mean (SD)	Group F *P*	Time F *P*	G × T F *P*	T1 vs. T2 T *P*	T1 vs. T3 T *P*	T1 vs. T4 T *P*
FIQ – Physical impairment	CBT	4.35 (2.12)	3.64 (2.30)	3.00 (2.43)	3.42 (2.29)				2.45 ns	0.71 ns	0.84 ns
	OBT	4.77 (2.24)	4.50 (1.91)	3.94 (2.06)	2.63 (1.57)				0.10 ns	0.63 ns	3.13 0.004
	AP	4.19 (2.08)	4.03 (2.09)	4.77 (2.60)	5.20 (2.51)				0.17 ns	-2.27 ns	-2.90 0.006
CBT vs. OBT (F, *P*)		1.28 ns	5.02 ns	1.73 ns	1.94 ns	3.07 ns	0.68 ns	6.57 0.001			
CBT vs. AP (F, *P*)		0.01 ns	1.28 ns	4.79 ns	6.36 0.010						
OBT vs. AP (F, *P*)		1.06 ns	0.76 ns	1.35 ns	16.38 <0.001						

Pain intensity	CBT	4.15 (0.84)	3.54 (1.03)	3.73 (0.94)	3.18 (1.42)				3.45 0.002	3.14 0.003	4.32 0.001
	OBT	4.22 (0.99)	4.12 (1.12)	3.77 (0.94)	3.05 (1.40)				0.56 ns	3.29 0.002	4.64 <0.001
	AP	3.80 (0.99)	3.79 (1.07)	4.07 (1.07)	4.14 (1.46)				0.39 ns	-2.82 0.008	-2.08 ns
CBT vs. OBT (F, *P*)		0.21 ns	2.23 ns	0.15 ns	0.49 ns	1.83 ns	11.70 <0.001	11.95 <0.001			
CBT vs. AP (F, *P*)		2.72 ns	1.04 ns	2.94 ns	8.77 0.004						
OBT vs. AP (F, *P*)		2.74 ns	1.69 ns	1.82 ns	11.79 0.001						

MPI – Affective distress	CBT	3.22 (1.02)	2.84 (1.10)	2.57 (1.05)	2.56 (1.24)				2.02 ns		
	OBT	3.17 (1.44)	3.31 (1.29)	3.14 (1.23)	2.92 (1.23)				-0.61 ns		
	AP	3.54 (1.29)	3.62 (1.34)	3.99 (1.37)	4.16 (1.44)				-0.90 ns		
CBT vs. OBT (F, *P*)		0.29 ns	2.43 ns	2.88 ns	1.09 ns	8.34 0.001	0.28 ns	5.89 <0.001			
CBT vs. AP (F, *P*)		1.96 ns	6.97 ns	20.65 <0.001	27.83 <0.001						
OBT vs. AP (F, *P*)		2.79 ns	0.96 ns	4.94 ns	9.26 0.004						

Number of physician visits	CBT	30.55 (16.20)			25.27 (18.47)						1.79 ns
	OBT	36.87 (15.15)			16.35 (18.26)						5.57 <0.001
	AP	34.13 (12.17)			47.65 (20.01)						-5.56 <0.001
CBT vs. OBT (F, *P*)		2.59 ns			4.77 0.010	10.99 <0.001	5.59 0.020	33.52 <0.001			
CBT vs. AP (F, *P*)		1.16 ns			24.22 <0.001						
OBT vs. AP (F, *P*)		0.72 ns			46.04 <0.001						

Comparisons refer to pre-treatment, post-treatment, and 6- and 12-month follow-ups in the CBT, OBT, and AP groups. ANOVA, analysis of variance; AP, attention-placebo; CBT, cognitive behavior therapy; FIQ, Fibromyalgia Impact Questionnaire; f/u, follow-up; MPI, Multidimensional Pain Inventory; ns, not significant; OBT, operant behaviour therapy; SD, standard deviation.

**Table 3:** Effect sizes of the dependent variables in the CBT and OBT groups in comparison with the AP group at pre-treatment (T1), post-treatment (T2), and 6 months (T3) and 12 months (T4) after treatment

Group								
	CBT – AP				OBT – AP			
Variable	ES T1–T2	ES T1–T3	ES T1–T4	ES T1–T4^a^	ES T1–T2	ES T1–T3	ES T1–T4	ES T1–T4^a^

FIQ – Physical impairment	0.18	0.83	0.84	0.71	0.21	0.37	**1.15**	**1.07**
MPI – Pain	0.29	0.40	**1.14**	0.31	0.33	0.30	**1.10**	**0.62**
MPI – Affective distress	0.76	**1.39**	**1.57**	**0.61**	0.22	0.59	0.86	**0.72**
PRSS – Coping	**1.16**	**1.72**	**2.66**	**1.70**	0.21	0.73	**1.23**	0.38
PRSS – Catastrophising	0.62	**0.96**	**1.44**	**0.54**	0.12	0.89	0.97	0.41
Pain behaviour	0.44	0.42	0.57	0.49	0.59	0.72	**1.89**	**1.48**
MPI – Solicitous spouse behaviour	0.19	0.41	0.44	0.37	0.43	**1.00**	**1.71**	**0.57**

^a^Adjusted ES based on the AP group with the dropout values from baseline carried forward to the 12-month follow-up. Medium (>0.5) and large (>0.8) ESs were bolded. AP, attention-placebo; CBT, cognitive behaviour therapy; ES, effect size; FIQ, Fibromyalgia Impact Questionnaire, MPI, Multidimensional Pain Inventory; OBT, operant behavior therapy; PRSS, Pain Related Self-Statements Scale.

**Table 4:** Means, SDs, and F and *P *values of the ANOVA effects for group, time, and group × time (G × T) and T and *P *values for secondary measures: cognitive variables and behavioural variables pre-treatment (T1) in comparison with post-treatment (T2), 6 months (T3) and 12 months (T4) in the CBT, OBT, and AP groups

Secondary variables – Cognitive variables											
						Main effects					
Secondary variables	Group	T1 Mean (SD)	T2 Mean (SD)	T3 Mean (SD)	T4 Mean (SD)	Group F *P*	Time F *P*	G × T F *P*	T1 vs. T2 T *P*	T1 vs. T3 T *P*	T1 vs. T4 T *P*
PRSS – Coping	CBT	3.25 (0.61)	3.68 (0.76)	3.74 (0.59)	3.89 (0.54)				-3.29 0.002	-3.28 0.002	-4.83 <0.001
	OBT	2.96 (1.02)	3.18 (0.99)	3.43 (0.80)	3.52 (1.10)				-1.82 ns	-3.43 0.002	-2.72 0.010
	AP	2.94 (0.68)	2.97 (0.99)	2.69 (0.72)	2.27 (0.99)				-0.37 ns	2.94 0.006	4.66 <0.001
CBT vs. OBT (F, *P*)		2.20 ns	5.90 0.02	3.29 ns	3.05 ns	20.44 <0.001	5.16 0.002	16.18 <0.001			
CBT vs. AP (F, *P*)		3.34 ns	18.01 ns	46.19 <0.001	74.50 <0.001						
OBT vs. AP (F, *P*)		0.01 ns	1.15 ns	17.28 <0.001	26.42 <0.001						
PRSS – Catastrophising	CBT	2.29 (0.93)	1.69 (1.04)	1.69 (1.08)	1.49 (1.06)				4.14 <0.001	3.55 0.001	4.12 <0.001
	OBT	2.48 (1.20)	2.13 (1.31)	1.67 (1.01)	1.67 (1.21)				2.48 0.018	4.65 <0.001	4.23 <0.001
	AP	2.36 (1.02)	2.27 (1.00)	2.58 (0.93)	2.83 (1.13)				1.05 ns	-2.37 0.023	-2.94 0.006
CBT vs. OBT (F, *P*)		0.54 ns	2.50 0.020	0.04 ns	0.44 ns	6.52 0.002	8.76 <0.001	12.99 <0.001			
CBT vs. AP (F, *P*)		0.10 ns	6.27 0.014	14.91 <0.001	27.54 <0.001						
OBT vs. AP (F, *P*)		0.19 ns	0.29 ns	16.25 <0.001	17.86 <0.001						
Secondary variables – Behavioural variables											
Pain behaviour	CBT	89.91 (73.40)	72.10 (56.63)	80.11 (65.79)	82.84 (73.32)				1.88 ns	0.97 ns	0.73 Ns
	OBT	114.16 (51.32)	73.96 (40.71)	64.89 (43.20)	42.63 (49.35)				2.67 0.011	3.03 0.005	3.43 0.001
	AP	88.79 (49.13)	104.38 (49.81)	111.1 (52.66)	124.4 (57.17)				-0.35 0.002	-3.39 0.002	-4.41 <0.001
CBT vs. OBT (F, *P*)		1.10 ns	0.03 0.02	1.23 ns	6.86 0.010	2.68 ns	2.37 ns	11.39 <0.001			
CBT vs. AP (F p)		0.01 ns	5.49 0.02	4.01 ns	5.94 0.020						
OBT vs. AP (F, *P*)		1.13 ns	7.01 0.01	13.73 <0.001	34.77 <0.001						
MPI – Solicitous spouse behaviour	CBT	3.30 (1.62)	2.97 (1.25)	3.32 (1.31)	3.39 (1.10)				0.68 ns	0.02 ns	-0.14 Ns
	OBT	4.00 (0.79)	2.95 (1.02)	3.20 (1.44)	2.76 (1.31)				4.33 <0.001	3.37 0.020	4.16 0.001
	AP	3.23 (1.25)	3.29 (1.21)	3.99 (1.17)	4.11 (1.16)				-0.68 ns	-2.91 0.006	-3.16 0.003
CBT vs. OBT (F, *P*)		2.43 ns	0.01 ns	0.02 ns	2.22 ns	1.18 ns	4.07 0.011	6.58 0.001			
CBT vs. AP (F, *P*)		0.01 ns	1.33 0.014	3.73 ns	3.25 ns						
OBT vs. AP (F, *P*)		3.07 ns	2.04 ns	4.34 ns	11.35 0.001						
